# Repeated influenza vaccination results in blunting rather than back-boosting of antibody responses against A(H3N2) antigens

**DOI:** 10.21203/rs.3.rs-10352604/v1

**Published:** 2026-07-31

**Authors:** Ziheng Zhu, Sheena G. Sullivan, Stephany Sánchez-Ovando, Leslie Dowson, A. Jessica Hadiprodjo, Louise Carolan, Vivian K.Y. Leung, David Hodgson, Christopher C. Blyth, Allen C. Cheng, Julia Clark, Kristine Macartney, Archana Koirala, Ameneh Khatami, Helen Marshall, Peter A.B. Wark, Adam J. Kucharski, Annette Fox

**Affiliations:** University of Melbourne; Royal Melbourne Hospital; University of Melbourne; University of Melbourne; Royal Melbourne Hospital; Royal Melbourne Hospital; Royal Melbourne Hospital; London School of Hygiene & Tropical Medicine; Telethon Kids Institute; Alfred Health; Queensland Children’s Hospital; The Children’s Hospital at Westmead; The Children’s Hospital at Westmead; The Children’s Hospital at Westmead; The Women and Children’s Health Network; John Hunter Hospital; London School of Hygiene & Tropical Medicine; Royal Melbourne Hospital

## Abstract

Hemagglutination inhibiting (HI) antibody responses against influenza A(H3N2) vaccine strains decline with increasing years of vaccination. It is hypothesized that memory B cell dominance may direct antibody responses towards epitopes conserved with previously-encountered vaccine strains, including non-HI epitopes. To investigate this, we compared healthcare workers with zero versus five consecutive years of vaccination prior to enrolment in 2020 or 2021. We evaluated HI antibody reactivity across 25 A(H3N2) viruses spanning 2007–2022 and antibody binding to recombinant hemagglutinins (HA). HI antibody responses were poor across strains for participants vaccinated five compared to zero of five prior years, independent of pre-vaccination titre. HA and HA-stem binding antibody titre-rises were likewise attenuated in the repeatedly vaccinated group. These results provide no evidence that repeated vaccination promotes antibodies targeting conserved epitopes, rather, repeated vaccination broadly attenuates antibody responses. Work is ongoing to investigate whether attenuation reflects deficient memory B cell differentiation into plasmablasts.

## Introduction

Influenza is a major vaccine-preventable respiratory disease that poses a persistent threat to global health^1,2^. The most widely administered vaccines comprise inactivated egg-grown viruses representing each influenza A subtype (A(H3N2) and A(H1N1)) and at least one influenza B virus. Ongoing antigenic evolution driven by the accumulation of point mutations and deletions in the viral genome (antigenic drift) necessitates frequent updates to influenza vaccine components and annual re-immunization^3–5^. Egg-based vaccines are estimated to be around 30–60% effective when vaccine strains are antigenically similar to circulating viruses^6–8^. Among seasonal influenza viruses, A(H3N2) undergoes the greatest antigenic drift^9,10^, corresponding to relatively poor vaccine effectiveness (VE)^7,8,11^. A review of studies published between 2004 and 2015 reported pooled VE of 33% against A(H3N2) compared to 67% for A(H1N1) and 54% for type B^6^. VE was lowest against A(H3N2) during the 2014–2015 influenza season when a T160K substitution in the hemagglutinin of circulating viruses introduced a glycosylation site near the receptor-binding domain, potentially shielding the virus from vaccine-induced antibodies and preventing them from binding to the HA^12,13^.

Prior influenza vaccination may also attenuate VE. Separate analyses from Mclean et al.^14^ and Skowronski et al.^15^ demonstrated that repeated prior vaccination was associated with reduced VE in some seasons, most notably during A(H3N2)-predominant years. Consistent with these findings, a systematic review of studies published between 2016 and 2022 reported an estimated 18% reduction in VE for A(H3N2) among individuals vaccinated in both the current and immediately preceding seasons, compared with those vaccinated in the current season only^16^. The effects of prior influenza vaccination vary across seasons, with beneficial versus attenuating outcomes reported. To account for this variability, the antigenic distance hypothesis proposes that “negative interference” that orients antibodies towards the first vaccine is greater when the first and second vaccine are more antigenically similar, and that negative effects on VE increase as the antigenic difference between circulating and vaccine strains increases^15,17^.

Early serological studies examining the effect of single prior vaccination on the immunogenicity of the subsequent influenza vaccination reported heterogenous outcomes^18,19^. However, more recent serological studies consistently report decreasing post-vaccination hemagglutination inhibiting (HI) antibody titres and titre-rises with increasing years of vaccination^20–22^. The immunological mechanisms underlying interference associated with prior influenza vaccination remain elusive. It has been established that responses to seasonally-updated influenza vaccines are dominated by memory B cells, some of which differentiate rapidly into antibody-secreting plasmablasts yielding a titre rise^23^. In turn, influenza-reactive plasmablasts induced by vaccination have highly mutated B cell receptors indicating that they are derived from memory B cells that have undergone somatic hypermutation^23^. This suggests that most antibody produced after seasonal influenza vaccination originates from memory B cells that were primed by past strains. Some studies suggest that memory B cells undergo additional somatic hypermutation and affinity mature to better recognize variant epitopes, ^23^ while others indicate that they maintain their priming epitope affinity^24,25^. Nevertheless, plasmablasts are predominantly produced within a week of vaccination, during which time they are unlikely to have undergone much B cell receptor mutation and affinity change. Consistent with this theory, exposure to A(H3N2) boosts antibodies against past strains for which pre-existing antibody is present, although the magnitude of this “back-boosting” declines with increasing antigenic distance from the encountered strain^26^. The antibody focusing hypothesis further proposes that memory B cells recognising epitopes conserved between prior and prevailing vaccine strains may outcompete naive B cells against more novel epitopes so that antibodies become focused towards more conserved epitopes^27^.

We established a cohort that recruited healthcare workers (HCWs) from multiple Australian hospitals across four years to investigate how vaccination experience during the preceding five years influences the immunogenicity and effectiveness of influenza vaccination. Analysis of the first two study years (2020 and 2021) shows that antibody responses against viruses representing both influenza A vaccine strains decline with successive years of annual vaccination^22^. Building on these observations, this study sought to determine whether repeated vaccination would drive antibody responses towards conserved epitopes shared with past strains, including antibodies that bind HA but lack HI activity. We analyzed a nested subset of cohort participants with contrasting vaccination histories (vaccinated none or all of the preceding five years, i.e. 0/5 or 5/5 prior years). Sera were titrated by HI against 25 A(H3N2) viruses including historical, recent and future vaccine strains. Additionally, enzyme-linked immunosorbent assay (ELISA) was used to quantify antibodies that bind recombinant HA proteins including full-length, head, and highly conserved stalk domain constructs.

## Results

### Cohort Demographics

The nested study recruited 151 HCWs across 2020 and 2021, of whom 142 provided pre-vaccination and post-vaccination blood samples and were eligible for analysis. 53/142 had been vaccinated 0/5 prior years (0-Prior) and 89/142 had been vaccinated 5/5 prior years (5-Prior) at enrolment in 2020 or 2021 (Table 1). 52/53 in the 0-Prior group and 51/89 in the 5-Prior group were included in HI antibody analysis. Of these 103 participants, 75 were enrolled in 2020 (0-Prior = 40; 5-Prior = 35) and 28 in 2021 (0-Prior = 12; 5-Prior = 16). Additionally, 25 participants enrolled in 2020 continued follow-up in 2021; 16 from the 2020 0-Prior group were reclassified as 1-Prior in 2021, and nine from the 2020 5-Prior group remained classified as 5-Prior. Although recruitment was not matched on age or sex; matching was applied only during selection of participants for serological testing and analysis. Despite attempts to select participants in the 5-Prior subset to be comparable to the 0-Prior group in terms of age and sex, demographic differences remained between these groups in 2021 (Table 1).

To enhance the statistical power, all 89 nested study participants in the 5-Prior group were included in ELISA analysis, which was restricted to day 0 and post-vaccination day 14 sera. In total, 394 samples collected from 142 HCWs during 2020 and 2021 were evaluated (Table 1). Blood-draws were performed between March and December of each study year. The distribution of sample collection relative to influenza vaccination is shown in Supplementary Figure S1.

### Pre-vaccination HI antibody reactivity across antigenically diverse A(H3N2) viruses comparing participants vaccinated 0/5 versus 5/5 prior years

**Figure 1:**
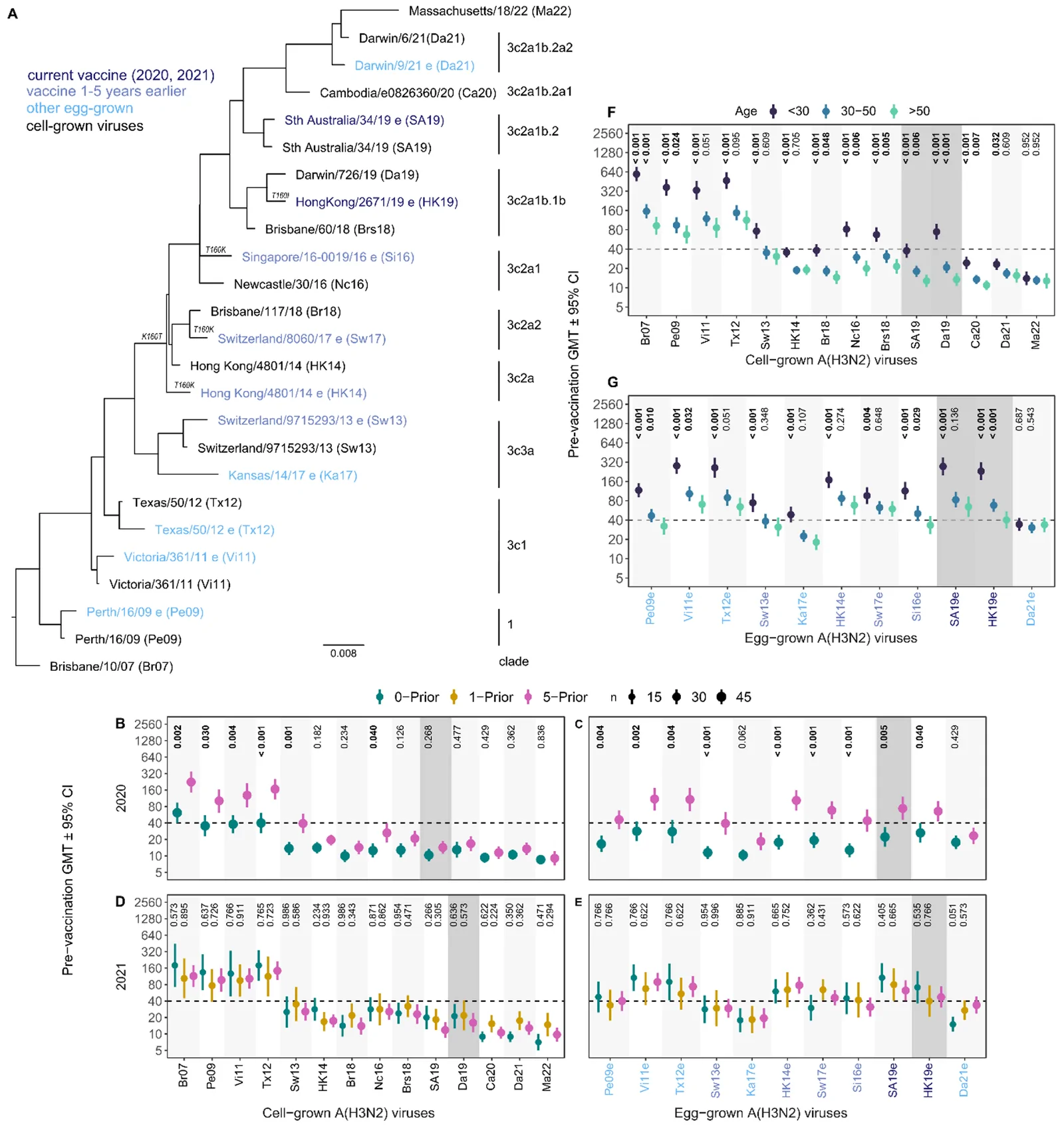
Pre-vaccination hemagglutination inhibition (HI) antibody titres against 25 influenza A(H3N2) viruses stratified by prior vaccination group, or age, and study year. (A) HA sequence phylogeny of A(H3N2) viruses in the HI antibody analysis panel. Viruses are categorized (see legend) as strains in vaccines administered to participants enrolled in 2020 and/or 2021; strains in vaccines administered up to 5 years prior to 2020 (2015-2019); other egg-grown viruses; and cell-grown viruses. An appended “e” denotes an egg-grown isolate. Abbreviated names are shown in parentheses. (B, D, F) Pre-vaccination geometric mean titres (GMTs) against 14 cell-grown viruses. (C, E, G) Pre-vaccination GMTs against 11 egg-grown viruses. Participants are stratified by vaccination history (B–E) or by age group (F, G). Points indicate GMTs for each virus and group, sized proportionate to the number of HCW contributing to the estimate. Vertical values above GMTs are p-values for t-tests comparing the 5-Prior group to 0-Prior or 1-Prior groups (B-E) or the youngest age group to the older age groups (F, G). P-values were adjusted for multiple comparison using the Benjamini-Hochberg false discovery rate procedure. P-values ≤ 0.05 are shown in bold. The dashed line marks the seroprotective threshold (HI ≥ 40). Dark grey vertical shading highlights 2020 and 2021 vaccine-like strains. Vertical bars around each point estimate show the 95% confidence interval. Virus names are abbreviated and coloured according to the scheme defined in panel A.

Sera were initially titrated by HI assay against a panel of 38 A(H3N2) viruses (Supplementary Table S1) dating back to 1968 when A(H3N2) emerged in humans. After testing sera from 50 participants, the panel was reduced to 25 viruses (14 cell-grown, 11 egg-grown) spanning 2007–2021 (Fig. 1A) because vaccination induced little to no rise in antibodies reactive against viruses preceding A/Brisbane/10/2007 (Supplementary Figure S2, S3).

In 2020, pre-vaccination (day 0) HI antibody profiles reflected vaccination history. The 5-Prior group had significantly higher geometric mean titres (GMTs) than the 0-Prior group against older cell-grown viruses (Fig. 1B) and against most egg-grown viruses (Fig. 1C). Differences were less pronounced against egg-grown A/Kansas/14/2017 (Ka17e) that had not been included in the Southern Hemisphere vaccine composition up until 2020, and against egg-grown A/Darwin/9/2021 (Da21e) that entered the vaccine composition in 2022 and had not previously been encountered by 2020 participants (Fig. 1C). On average 5-Prior participants had pre-vaccination seropositive titres (HI ≥ 40) against 44% of cell-grown and 62% of egg-grown viruses, whereas 0-Prior participants were seropositive against only 25% of cell-grown and 29% of egg-grown viruses (Supplementary Table S2). In contrast in 2021, pre-vaccination GMTs were similar between prior vaccination groups (Fig. 1D, E). Notably, the 0-Prior group enrolled in 2021 was younger than other groups (Table 1), with birth years ranging from 1994 to 2003. Stratification by age demonstrated that pre-vaccination GMTs against viruses spanning 2007 to 2020 were highest amongst the youngest participants (Fig. 1F,G) regardless of vaccination history (Supplementary Figure S4). For both prior vaccination groups and years, antibody titres were lower against cell-grown viruses circulating since 2014 compared to earlier viruses (Fig. 1B,D).

### Post-vaccination HI antibody reactivity across A(H3N2) viruses comparing participants vaccinated 0/5 versus 5/5 prior years

**Figure 2:**
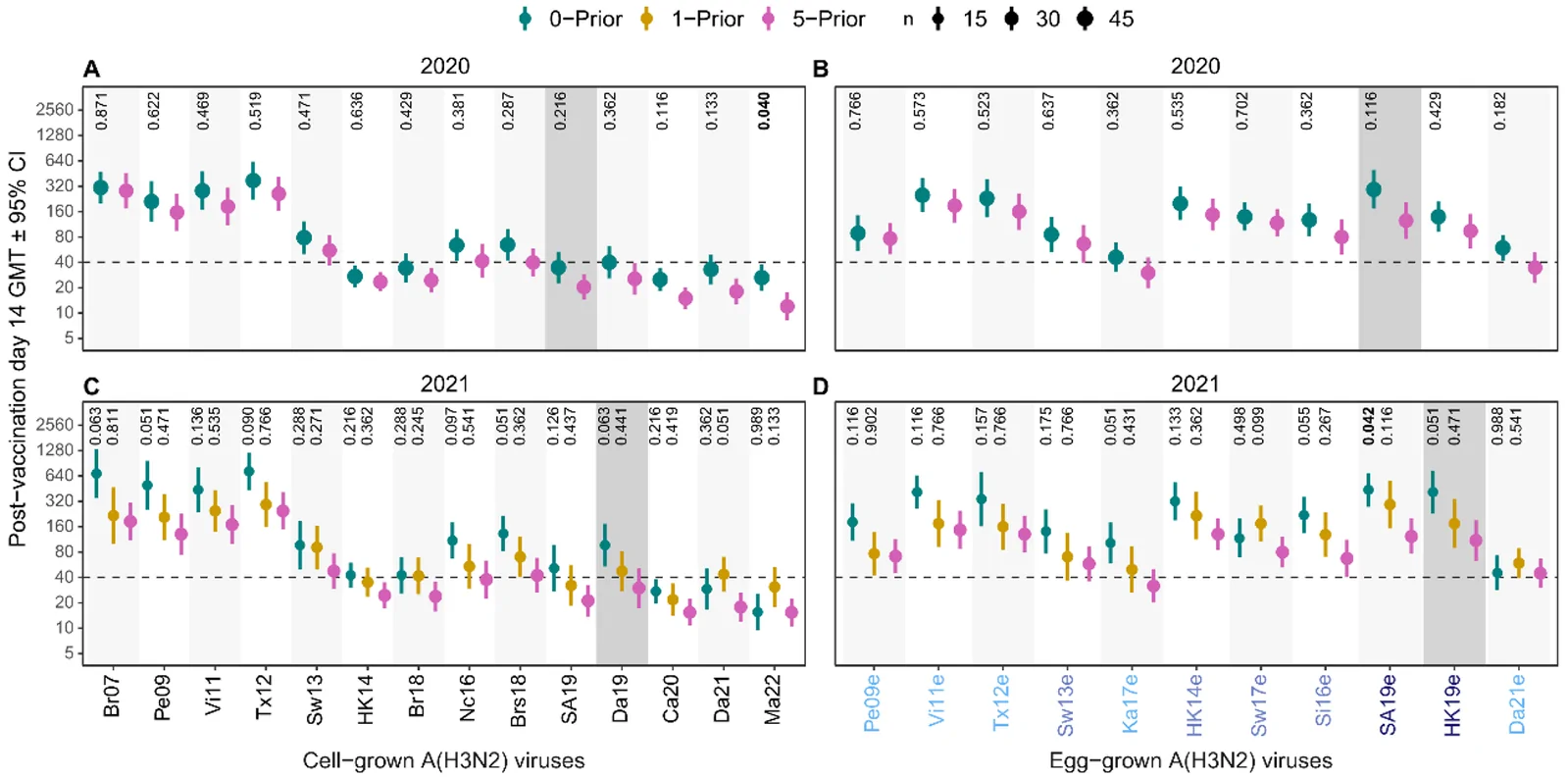
Post-vaccination HI antibody titres against 25 influenza A(H3N2) viruses stratified by prior vaccination group and study year. Post-vaccination GMTs against 14 cell grown viruses (A,C) and 11 egg-grown viruses (B,D). HCWs were enrolled in 2020 and vaccinated 0/5 or 5/5 prior years (A,B) or in 2021 and vaccinated 0/5, 1/5, or 5/5 prior years (C,D). Points indicate GMTs for each virus and group, sized proportionate to the number of HCW contributing to the estimate; vertical bars around each point estimate show the 95% confidence interval. Vertical values above GMTs are p-values for t-tests comparing the 5-Prior group with 0-Prior and 1-Prior groups and are adjusted for multiple comparison using the Benjamini-Hochberg false discovery rate procedure. P-values ≤ 0.05 level are shown in bold. The dashed horizontal line marks the seroprotective threshold (HI ≥ 40). Dark grey vertical shading highlights 2020 and 2021 vaccine-like strains. Virus names are abbreviated and coloured according to the scheme defined in panel Figure 1A.

By ~ day 14 after vaccination, the 0-Prior group showed higher GMTs compared to the 5-Prior group across multiple viruses, though few differences were statistically significant (Fig. 2, Supplementary Table S2). In 2020, this included the cell-grown virus (Da21) that emerged after the vaccine strain A/South Australia/34/2019 (SA19) (Fig. 2A,B). In 2021, participants in the 0-Prior group had higher GMTs across a broad range of strains spanning 2007–2020 and including the prevailing vaccine strain (A/Hong Kong/2671/2019 (HK19e)) (p = 0.051), with statistical significance reached against only the egg-grown SA19e (Fig. 2C,D).

### Association between pre- and post-vaccination HI antibody titres across prior vaccination groups

**Figure 3:**
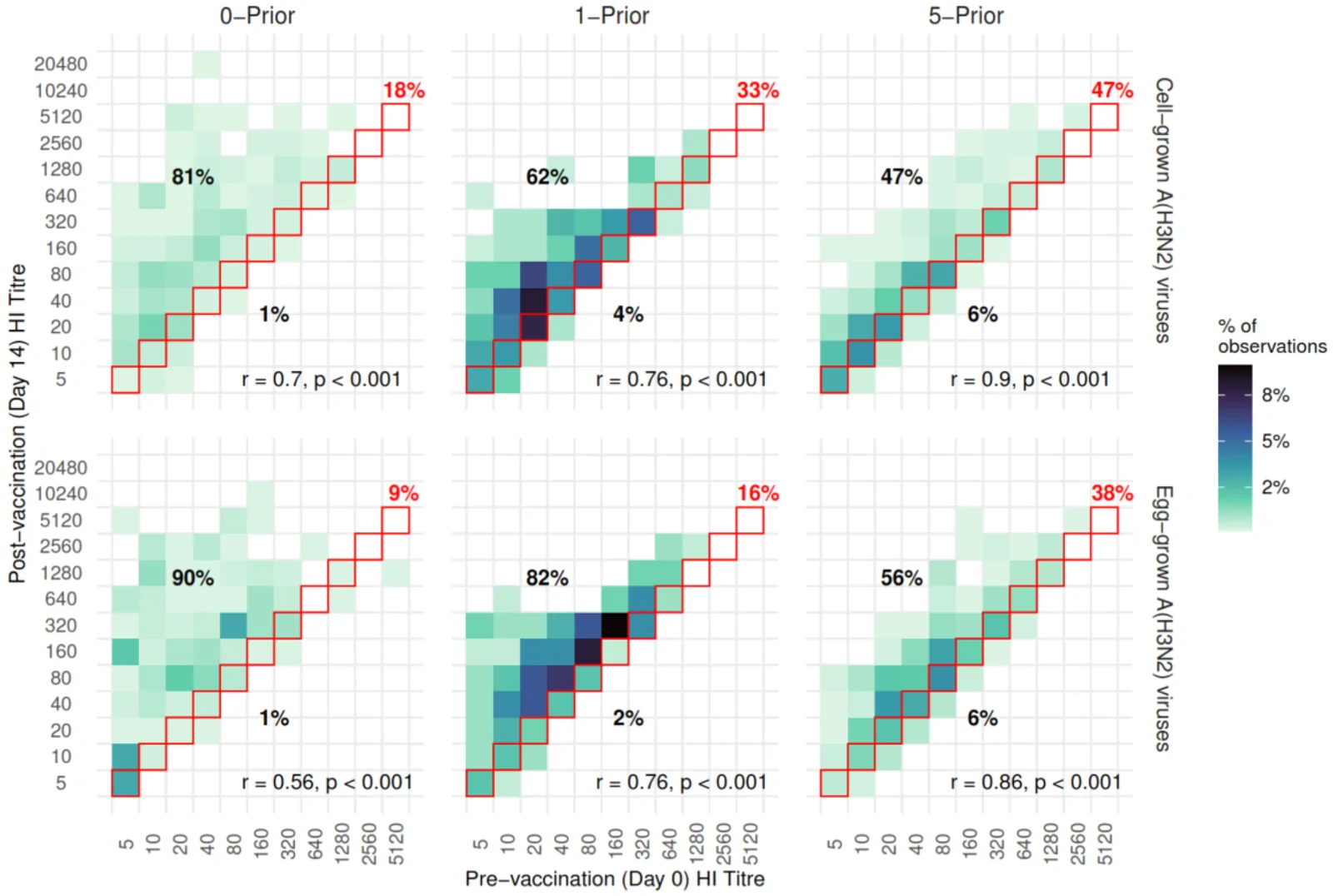
Relationship between pre-vaccination and post-vaccination HI antibody titres stratified by prior vaccination group. Heatmaps display the frequency of observations for pre-vaccination (day 0) versus post-vaccination (day 14) HI titres against 14 cell-grown viruses (Top panels) and 11 egg-grown viruses (Bottom panels). Data for the 2020 and 2021 cohorts are combined: 0-Prior (n = 52 titre pairs/virus), 1-Prior (n = 16 titre pairs/virus) and 5-Prior (n = 60 titre pairs/virus). Text in red indicates the percentage of observations showing no change in titre (red diagonal squares), whilst the upper left and lower right values represent the percentages showing titre rise and titre decline by day 14, respectively. Spearman’s correlation coefficients (r) and associated P-values (p) are shown at the bottom right of each facet.

Given that pre-vaccination titres varied across prior vaccination groups, we next examined relationships between pre- and post-vaccination titres. Figure 3 demonstrates that at each pre-vaccination titre, post-vaccination titres were more clustered on, or near the diagonal (no titre change) for the 5-Prior group relative to the 0-Prior group. For the 5-Prior group, the percentage of titre pairs on the diagonal was 47% for cell-grown viruses and 38% for egg-grown viruses, and a relatively large fraction of titre pairs were below 80 and clustered on or near the diagonal. For the 0-Prior group, the percentage of titre pairs on the diagonal was only 18% for cell-grown viruses and 9% for egg-grown viruses, and for egg-grown viruses, titres of 5 were relatively clustered on the diagonal. The relationship for the 1-Prior group was intermediate between 0-Prior and 5-Prior groups in terms of titre pairs on the diagonal and correlation coefficients. Consistent with effects on post-vaccination titres, pre-vaccination HI titres were inversely associated with titre rise at day 14 against most viruses for the 0- or 1-Prior groups, but not the 5-Prior group (Supplementary Figure S5).

### HI antibody titre rise across A(H3N2) viruses by prior vaccination group, pre-vaccination titre and age

To further investigate the effect of repeat prior vaccination on day 14 post-vaccination titre rise, we fitted a mixed-effects model across all 25 A(H3N2) viruses, with log-transformed pre- to post-vaccination titre fold-change as the outcome. Prior vaccination group was the primary predictor with the 5-Prior group as the reference. Virus strain, sample year, sex, and age were included as covariates, and participant specific random intercepts were used to account for repeated measurements across viruses. Given the potential influence of pre-vaccination antibody levels on subsequent titre rise (Fig. 3), an interaction between prior vaccination history and pre-vaccination titre was also included. Model fit was evaluated using likelihood ratio tests, Akaike Information Criterion (AIC) and Bayesian Information Criterion (BIC) values across candidate models.

**Figure 4:**
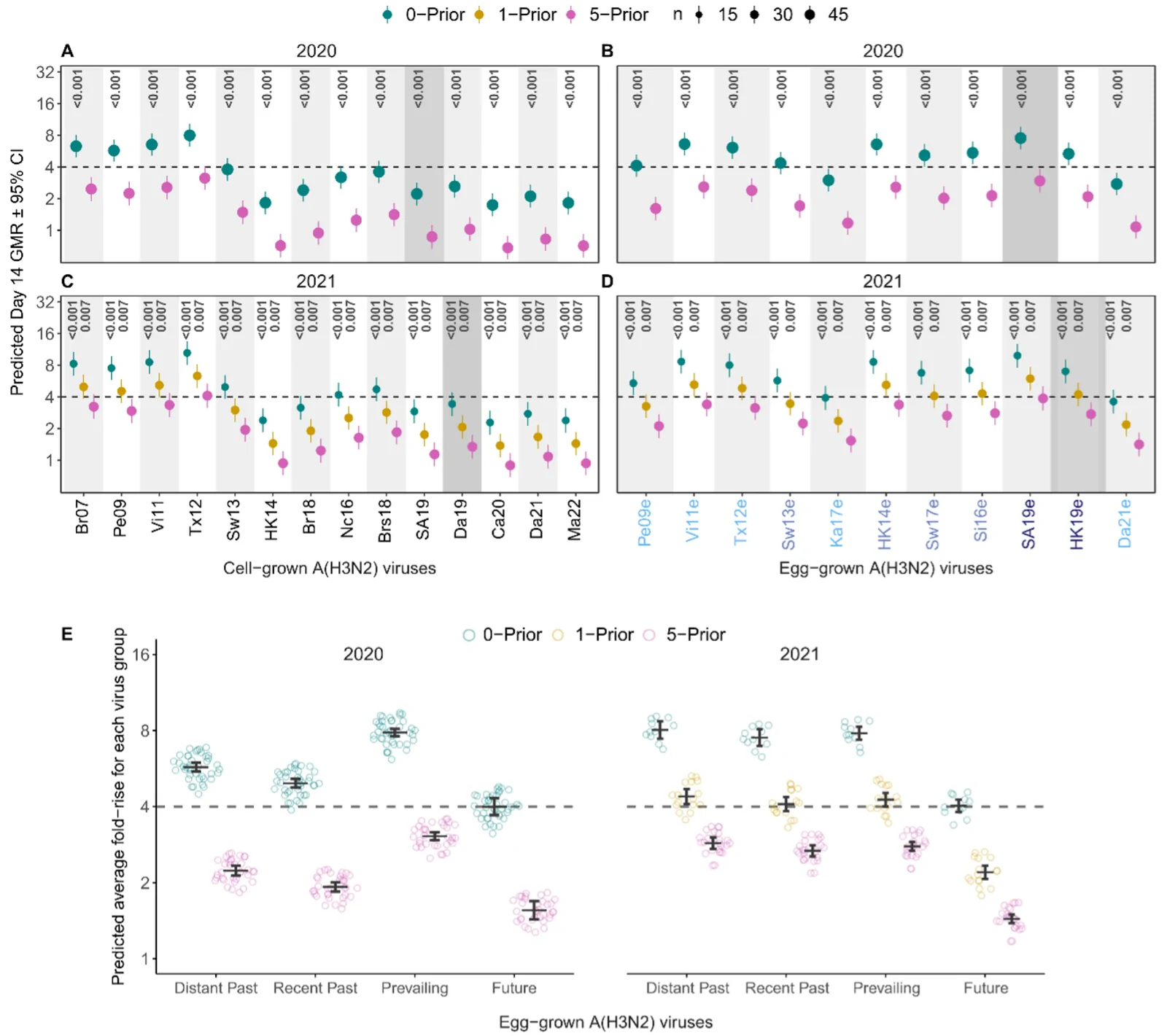
Estimated post-vaccination HI antibody fold-rises across cell-grown and egg-grown A(H3N2) viruses stratified by prior vaccination group and study year. (A–D) Predicted day 14 post-vaccination GMRs (points) with 95% confidence intervals (vertical bars) from the mixed-effects linear regression model, separated by study year and virus propagation (egg- or cell-grown). Point sizes are proportionate to the number of participants contributing to the estimate. Virus names are abbreviated and coloured according to the scheme defined in Figure 1A. Dark grey vertical shading indicates vaccine like strains. P-values for linear contrasts comparing the 0-Prior and 1-Prior groups to the 5-Prior group are shown above each estimate and were adjusted for multiple comparison using the Benjamini-Hochberg false discovery rate procedure. (E) Average model-predicted fold-rises against egg-grown viruses grouped by vaccine season categories (Distant Past, Recent Past, Prevailing, Future). Values for each individual (open circles) are shown together with geometric means and 95% confidence intervals. Dashed horizontal lines at fold-rise=4 in all panels indicate the seroconversion threshold.

Estimates presented in Table 2 indicate that prior vaccination affects the fold-rise in post-vaccination titre independent of pre-vaccination titres. Post-vaccination titre fold-rise was estimated to be 2.6-fold (95% CI: 1.9–3.4) greater for the 0-prior compared to the 5-Prior group. A similar, though smaller, positive effect was observed for the 1-Prior group compared to the 5-Prior group, with fold-rise estimates of 1.5-fold (95% CI: 1.1–2.1). Pre-vaccination titre was negatively associated with post-vaccination titre rise, with each doubling of pre-vaccination titre associated with a 32% reduction in fold-rise (ratio: 0.68, 95% CI: 0.66–0.71). Day-14 fold-rises predicted from the model were substantially lower for all viruses for the 5-Prior compared to the 0-Prior group in 2020 (Fig. 4A,B), and in 2021 (Fig. 4B,C).

To assess cross-reactivity, we calculated for each participant the proportion of viruses for which the predicted fold-rise met the seroconversion threshold, analysing the 14 cell-grown and 11 egg-grown viruses separately. These individual level proportions were then averaged for participants within each prior vaccination group. In 2020, the 0-Prior group seroconverted against 51% (95%CI: 39–63) of cell-grown and 73% (95%CI: 62–84) of egg-grown viruses on average. In contrast, the 5-Prior group seroconverted against 3% (95%CI:0–5%) of cell-grown and 6% (95%CI:0–13%) of egg-grown viruses on average. Similarly, in 2021, participants in the 0-Prior group seroconverted against higher percentages of viruses than the 5-Prior group (Supplementary Table S3).

Participant was included as a random intercept to account for repeated measures across viruses.

### Distribution of the HI antibody responses against viruses grouped by circulation period relative to the prevailing vaccine strain

To investigate whether repeated prior vaccination favours the production of antibodies that target past strains suggestive of memory B cell recall and dominance, egg-grown viruses were categorised based on when they were included as vaccine strains relative to the prevailing vaccine strain. One group comprised viruses in vaccines administered in the past 5 years (Recent Past) that would have been encountered by the 5-Prior group. Otherwise, viruses were categorized as Distant Past, Prevailing, and Future vaccine strains (Fig. 4E; Supplementary Table S4). The 5-Prior group did not demonstrate greater titre rise against Distant or Recent Past vaccine strains in either 2020 or 2021. In 2020, titre rise was similarly focused on the Prevailing vaccine strain for both the 5-Prior and 0-Prior groups. In 2021, titre rise was relatively even across Prevailing and Past vaccine strains in all prior vaccination groups, and uniformly lower against Future strains.

### HA-binding IgG titres by prior vaccination group

HI antibodies primarily target epitopes adjacent to the receptor binding site in the variable head of the HA protein. To examine whether repeated vaccination may focus antibodies on highly conserved epitopes, which predominate in the HA stem region, we measured HA binding IgG titres via ELISA.

**Figure 5:**
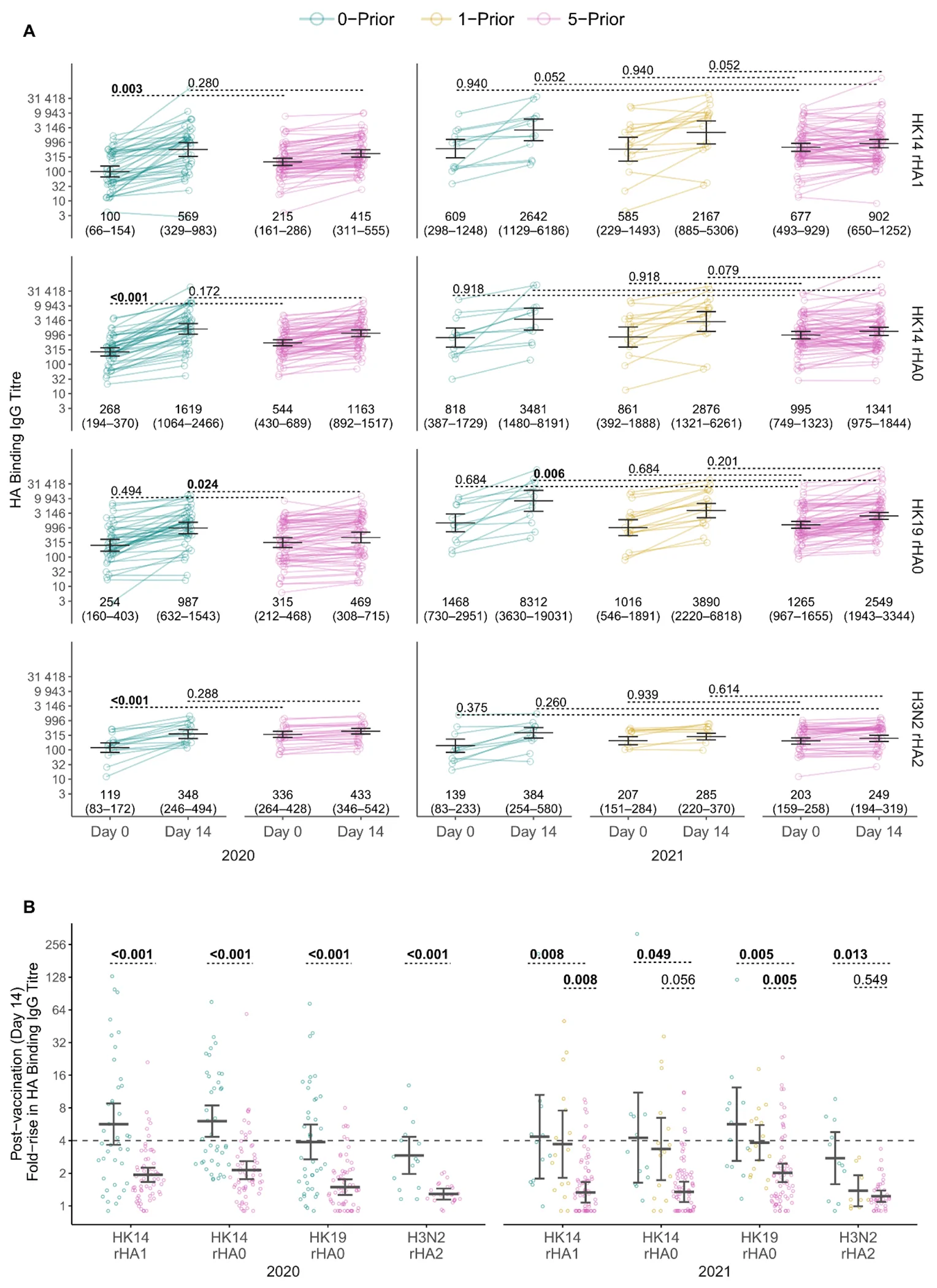
A(H3N2) hemagglutinin (HA) binding antibody responses stratified by prior vaccination group and study year. (A) Binding antibody titres are shown for four different HA constructs. Data are separated by prior vaccination group and study year. Open circles show data for each participant with lines connecting results for day 0 and day 14. Geometric mean titres and 95% confidence intervals are indicated by black horizontal bars and error bars, with values shown below each group. (B) Fold-rises from day-0 to day-14 in binding titres for each HA construct. Open circles are results for each participant. Geometric means and 95% confidence intervals are indicated by black horizontal bars and error bars. P-values shown above 0-Prior or 1-Prior groups in all panels are for unpaired t-tests compared with the 5-Prior group and were adjusted using the Benjamini-Hochberg false discovery rate procedure. P-values ≤ 0.05 are shown in bold.

Figure 5A show pre-vaccination and day 14 post-vaccination IgG binding titres against full-length (HA0), HA head (HA1) and HA stem (HA2) constructs. Pre-vaccination binding titres showed similar trends to HI antibodies. In 2020, binding titres were higher for the 5-Prior group compared to the 0-Prior group except for HA of A/Hong Kong/2671/2019 (HK19), which had not yet been included as a vaccine strain. In 2021, pre-vaccination HA-binding titres were not significantly different between prior vaccination groups. By day 14 post-vaccination binding titres were at least as high for the 0-Prior group compared to the 5-Prior group, and in 2021, titres against HA of HK19 were significantly higher for the 0-Prior group. Accordingly, binding titres rose significantly more after vaccination for the 0-Prior group compared to the 5-Prior group, in both 2020 and 2021 (Fig. 5B; Supplementary Table S5). Binding titres also rose significantly more for the 1-Prior group compared to the 5-Prior group, except for HA2. IgG binding titres against HA2 constructs were positively correlated with binding titres against swine HA0 (Supplementary Figure S6), supporting the use of the HA2 constructs as a probe to quantify HA stem directed antibody responses. Although HA2 binding antibody titres were relatively high for the 5-Prior group compared to the 0-Prior group at the pre-vaccination time-point, titre rise was relatively low indicating that repeated vaccination does not preferentially induce antibodies targeting conserved stem epitopes.

## Discussion

This study investigated why influenza vaccine immunogenicity against A(H3N2) viruses diminishes with increasing years of vaccination and whether repeated vaccinations direct antibodies towards epitopes that are relatively conserved with past strains. We found that vaccination-induced HI antibody titre rise was broadly attenuated across the A(H3N2) strains tested among repeatedly vaccinated HCWs studied here. Attenuation remained evident after adjusting for pre-vaccination titres, age and sex, and was pronounced in both 2020 and 2021, confirming that reduced antibody production cannot be explained solely by pre-existing antibody. We found no evidence that repeated prior vaccination promoted preferential boosting of HI antibodies against past over prevailing strains. Similarly, there was no evidence that repeated vaccination drove antibody production towards more conserved HA epitopes that could be detected by ELISA but not by HI assay.

Vaccination induced relatively little rise in antibodies that bind full-length HA or the relatively-conserved HA2/stem domain among repeatedly vaccinated HCWs. This is contrary to the hypothesis that successive exposures to variant vaccine strains may drive antibody responses towards conserved epitopes due to competitive memory dominance^27^, but could reflect sub-dominance of the HA stem. Other studies report that far fewer HA-reactive memory B cells target the stem compared to the head^28,29^. This HA stem sub-dominance has been attributed to poor accessibility of the HA stem and to inherent poly-reactivity of antibodies that bind to poorly accessible epitopes^29^. Consequently, HA-head binding memory B cells are preferentially activated to differentiate into antibody secreting plasmablasts, whereas HA stem-binding memory B cell activation mainly occurs when HA head reactive memory is absent^29^. Nevertheless, the poor HI antibody responses of repeatedly vaccinated HCWs suggests that HA head-binding memory B cell activation is also poor. A possible explanation is that memory B cells elicited by prior strains bind mutated HI epitopes in the current strain with low affinity that is enough to monopolize antigen and suppress de novo responses but too weak to trigger differentiation into plasmablasts.

Overall, our results align with previous studies showing that elevated pre-vaccination antibody titres can blunt serological responses following re-exposure^11,18^. However, for the 5-Prior group titre rise was similarly low across pre-vaccination titres suggesting that other components of pre-existing immunity such as memory B cells or T cells act in addition to, or in concert with pre-existing antibodies. Mechanistically, circulating antibodies may limit B cell re-activation by promoting antigen clearance via Fc-receptor-mediated uptake by phagocytic cells, or by masking cognate and nearby epitopes^30^. The extent to which antibody masking impacts responses against more distant epitopes through steric hindrance is debated^29,31,32^. Additionally, antibodies in complex with antigen, can prevent B cell activation by co-engaging inhibitory FcyRIIb receptors (CD32b) when a B cell recognizes antigen via the B cell receptor^33^. B cells with high BCR affinity for antigen require a larger inhibitory signal to be silenced^34^. Therefore, the effect of pre-existing antibodies may vary depending upon the range of memory B cell affinities for antigen. Pre-existing antibodies could potentially have a greater dampening effect on memory B cells elicited by older vaccines, depending on the extent to which epitopes in prevailing vaccine strains have mutated.

Titration of HI antibodies against 25 A(H3N2) viruses spanning 2007 to 2022 yielded some expected patterns. First, repeatedly vaccinated participants had higher pre-vaccination titres against previously encountered vaccine strains. Second, titres were low against cell-grown viruses in the clade 3c2a lineage due to the addition of an N-linked glycosylation site in a prominent antigenic site of HA^35^. Finally, younger individuals, whose primary exposures were to more recent strains, had higher pre-vaccination titres to viruses circulating since 2007, aligning with the concept of antigenic seniority, whereby antibody titres are typically higher against virus strains encountered earlier in life^36,37^. We found that HCWs in the 0-Prior group seroconverted to a wider range of viruses after vaccination than those who had been repeatedly vaccinated. It is difficult to ascertain whether this could reflect the recruitment of memory B cells specific for many past strains or cross-reactivity of a smaller set of antibodies at high concentration. Antibody binding to antigen is reversible. Therefore, at low antibody concentrations, only high-affinity interactions may reach sufficient occupancy to be detected via HI assay whereas at high antibody concentrations, even low affinity interactions with variant epitopes may be detected. Some evidence of cross-reactivity comes from titre-rises against strains such as A/Darwin/9/2021, which had not been used in the vaccines received by these HCWs and had not circulated widely. Given minimal influenza circulation during 2020 and 2021 due to COVID-19 restrictions, these responses were unlikely to reflect recent infection and the presence of memory B cells elicited by A/Darwin/9/2021. Further work is underway to assess B cell receptor gene use by HA-reactive B cells to understand antibody responses at the clonal level.

Several factors limit the statistical power, generalisability and interpretation of our results. We focused on healthcare workers because vaccination is relatively common in this group compared to other healthy adults. However, this made it difficult to recruit participants lacking prior vaccinations, particularly in 2021. The method used to quantitate the breadth of HI antibodies (percentages of viruses against which participants were seropositive or seroconverted) may be too simplistic. A logical scale for placing viruses along an x-axis is required for the approach of fitting titre landscapes across viruses to calculate area under the curve or the antigenic focus of the antibody response^38^. Arrangement of viruses by year of circulation is problematic due to the circulation of distinct genetic clades in the same year such as A/Switzerland/8060/2017 from clade 3c2a2 and A/Kansas/14/2017 from clade 3c3a (Fig. 1A). Genetic distances between viruses do not directly translate to antigenic distances, and antigenic cartography requires antisera to be generated in influenza naïve ferrets, which is costly.

## Conclusion and Implications

In summary, we found that serological responses against A(H3N2) viruses were broadly attenuated among repeatedly vaccinated HCWs studied here. Our results do not support the hypothesis that memory B cell dominance focuses responses on epitopes conserved with past strains. Rather, they suggest that memory B cell activation and differentiation into plasmablasts may be poor among repeat compared to first time vaccinees. Attenuation may in part be intrinsic to the memory B cell repertoire induced and maintained through successive vaccinations because attenuation was observed when pre-existing antibody titres were low. Studies of B cells within peripheral blood mononuclear cell (PBMC) samples from these participants are underway to investigate the magnitude, clonal dynamics and repertoire of memory B cell responses.

## Methods

### Study Design – participant selection and investigation

This study was nested within a larger cohort HCWs described by Sullivan et al^22^. Briefly, HCWs were recruited between 2020 and 2023 from tertiary hospitals across five states in Australia with a target enrolment of 250 HCWs per site. At enrolment, participants reported their influenza vaccination status for each of the preceding 5 years. Blood samples for serum preparation were collected before vaccination and approximately 14-days, and 6-months after vaccination. Those vaccinated 0/5 prior years (0-Prior) or 5/5 prior years (5-Prior) were invited to participate in a nested study to investigate immunological mechanisms mediating effects of repeated vaccination. Nested study participants had an additional blood draw 7-days after vaccination and provided additional blood tubes for peripheral blood mononuclear cell preparation at each time-point. These additional samples are not discussed in this study. The target sample size for the nested study was 60 per vaccination group, corresponding to 10 participants per vaccination group per site each year. However, this target was not reached for the 0- Prior vaccination group. Participant recruitment was not matched on age or sex; rather, matching was applied during selection of the subset included in the serological analyses. Specifically, all eligible nested study participants vaccinated 0/5 prior years were selected, and a 1:1 age and sex matched subset of participants vaccinated 5/5 prior years was selected using nearest-neighbour propensity score matching implemented in the MatchIt package^39^ in R. Propensity scores were estimated using a generalised linear model including age as a continuous variable and sex. Participants selection was restricted to those with serum samples collected pre-vaccination (day 0) and day 14 post-vaccination.

### Ethical Approvals

Ethical approval for the HCW study including nested sub-studies was granted by the Research Ethics Committee of the Royal Melbourne Hospital (HREC Reference Number: HREC/54245/MH-2019). All study staff were trained in Good Clinical Practice and Human Subjects Protection. All participants provided written informed consent, and the study was conducted in accordance with institutional and national ethical guidelines.

#### Hemagglutination inhibition (HI) assay

Sera were titrated by HI assay against a panel of A(H3N2) viruses to investigate whether repeated prior vaccination directs antibody responses towards past over prevailing strains. The initial panel comprised 38 viruses (Supplementary Table S1) dating back to 1968 when A(H3N2) emerged in humans but was reduced to 25 viruses spanning 2007–2021 (Fig. 1A) after initial testing. The final panel includes 11 egg-grown viruses used as vaccine strains between 2009 and 2022, their cell-grown equivalents and three additional cell-grown viruses representing other recent clades not included in vaccines. HI assays were performed as described previously ^40^. In brief, sera were treated with receptor destroying enzyme (Denka Seiken) to remove non-specific hemagglutination inhibitors, adsorbed with 20% guinea pig red blood cells to remove non-specific agglutination, then diluted two-fold from 1:10 to 1:10240 (25 μl/dilution) in U-bottom 96-well microplates. Four hemagglutination (HA) units/25 μl of virus was added and incubated for 30 minutes at room temperature followed by 25 μl or 1% guinea pig red blood cells incubated for 90 minutes. HI antibody titres were read using an automated reader (Cypherone, InDevR, Colorado, USA) and recorded as the reciprocal of the highest serum dilution causing complete inhibition of agglutination^22,41^.

#### Hemagglutinin (HA) binding antibody titration

ELISA was performed to determine serum IgG binding titres against five recombinant HA (rHA) proteins of A(H3N2) viruses. Full-length HA (HA0) of A/Hong Kong/2671/2019 (HK19) (Cat#40721-V08H; accession number: EPI1694849, Sino Biological, Beijing, China) the 2021 vaccine antigen, served as the prevailing strain antigen. A/Hong Kong/4801/2014 (HK14) proteins were included to assess binding to recent past strains (HA0 Cat#40555-V08B and HA1 Cat#40555-V08H; accession number: EPI653201, Sino Biological). Full-length HA of A/swine/Colorado/A01203748/2012 (Co12) (Cat#40689-V08H; accession number: AFU10042) and A/Wisconsin/12/2010 (Wi12) (Cat#FR-991 International Reagent Resource, Manassas, VA, USA) were used to quantify antibodies with broad recognition across the H3N2 HA. A recombinant H3N2 HA2 subunit (kindly provided by A/Prof Adam Wheatley) was used to quantify antibodies targeting the conserved stem.

Flat-bottom 96-well MaxiSorp plates (Thermo Fisher Scientific, Waltham, MA, USA, Cat# 439454) were coated with HA1 and HA2 subunit proteins at 25 ng/well and with full-length HA proteins at 40 ng/well in PBS and incubated overnight at 4°C. Plates were washed three times with PBS containing 0.05% Tween-20 (PBS-T) and blocked with 200 μl/well of blocking solution (PBS-T with 5% skim milk) for 1 hour at room temperature. Sera were serially diluted in half-log_10_ increments starting at 1:10 in sample diluent (PBS-T with 2% skim milk). A mouse monoclonal antibody against rHA of A/Victoria/361/2011(H3N2) (IRR, Manassas, VA, USA; Cat#FR-1122) was used as a standard and serially diluted 2-fold from 1:500 in sample diluent. Diluted serum and standard antibody (100 μl/well) were added and incubated for 1 hour at room temperature. After six washes with PBS-T, 100 μl/well of horseradish peroxidise (HRP)-conjugated goat anti-human IgG (1 mg/ml; Invitrogen, Carlsbad, CA,USA; Cat#62-8420) diluted in 1:1000 with sample diluent for test sera and anti-mouse IgG-HRP (Bio-Rad, Hercules, CA, USA; Cat#1721011) diluted in 1:1000 with sample diluent for the standard. Plates were incubated for 1 hour at room temperature, washed six times, and developed with 100 μl/well of room temperature 3,3′,5,5′-Tetramethylbenzidine (TMB) substrate (BD Biosciences, San Jose, CA, USA). The reaction was stopped after 10 minutes with 100 μl/well of 1M HCl and optical density was measured at 450 nm using SPECTROstar microplate reader. Absorbance values were fitted against dilution factors using a sigmoidal dose-response curve model in GraphPad Prism (Version 10, San Diego, CA, USA), with the bottom of the curve constrained to be greater than 0 and Hill Slope constrained to be greater than 1. Titres were interpolated as the reciprocal serum dilution at which 50% of the maximum binding was achieved.

### Statistical analysis

HI and HA binding titres were log transformed for all statistical analyses and subsequently back transformed to titre values for interpretation. HI titres of < 10 were assigned a value of 5. For the single sample that reach the upper limit of 10,240, a value of 20,480 was assigned for calculation purposes. Crude geometric mean titres (GMTs) and geometric mean ratios (GMRs) were calculated by prior vaccination status and virus or protein. Seropositivity was defined as a HI titre ≥ 40. For each participant, we calculated the proportion of 14 cell-grown or 11 egg-grown viruses for which seropositivity was achieved at each timepoint. These individual level proportions were then averaged within each prior vaccination group and reported with 95% confidence intervals. Between-group comparisons were assessed using unpaired t-tests, while within-group comparisons were assessed using paired t-tests. P-values were adjusted using Benjamini-Hochberg false discovery rate method. Spearman’s rank correlation coefficients were calculated to determine the strength and direction of associations between pre- and post-vaccination HI antibody levels, and between HI and HA binding antibody titres.

A linear mixed-effects model was fitted using the *lmer* function in the lme4 package^42^ to evaluate factors associated with log-transformed HI antibody fold-rises by day 14 post-vaccination. The model included prior vaccination group, virus strain, study year, sex, age and log-transformed pre-vaccination titre as fixed effects. Virus strain included all 25 A(H3N2) viruses. Prior exposure group, virus strain, sex and study year were included as categorical variables, with the 5-Prior group, SA19e, female and the 2020 study year used as the respective reference categories. An interaction term between prior vaccination group and log-transformed pre-vaccination titres was included to assess whether the relationship between pre-vaccination titre and post-vaccination fold-rise differed by vaccination history. Pre-vaccination HI titres and age were centred to their respective sample means before model fitting to improve interpretability of the intercept and interaction terms. A random intercept per participant was included in the model to account for repeated measures across virus strains. Model selection was performed by comparing AIC and BIC values from likelihood ratio test, with the model including the interaction term selected as the best-fitting model based on lowest AIC. Sex and age were retained in the model to avoid residual confounding after matching^43^. Predicted day 14 post-vaccination GMRs and 95% confidence intervals per virus were estimated using emmeans package^44^. P-values for comparisons between prior vaccination groups were obtained from emmeans contrast and adjusted for multiple testing using Benjamini-Hochberg false discovery rate methods.

For each participant-virus pair, predicted fold-rises were used to classify seroconversion (≥ 4-fold). For each participant, we then calculated the proportion of 14 cell-grown and 11 egg-grown viruses for which seroconversion was predicted. These participant proportions were then averaged by prior vaccination group and presented as means with 95% confidence intervals.

To investigate whether repeated vaccination promotes the production of antibodies that target past strains, egg-grown vaccine strains were grouped by year(s) that they were included as vaccine strains relative to the current vaccine. The Recent Past group comprised viruses in vaccines administered up to 5 years prior; otherwise, viruses were categorized as Distant Past, and Future vaccine strains. Model-predicted fold-rises were used to calculate average fold-rises for each virus-group and participant, which were then averaged by prior vaccination group and presented as means and 95% confidence intervals.

All analyses were performed in R version 4.4.2^45^, with *p* ≤ 0.05 considered statistically significant.

## Supplementary Material

This is a list of supplementary files associated with this preprint. Click to download.

• SupplementaryMaterial.docx

## Figures and Tables

**Table 1:** Baseline demographic characteristics of eligible nested study participants and the subset included in HI analysis.

	2020			2021		
	Group	0-Prior	5-Prior	0-Prior	1-Prior	5-Prior
**Newly enrolled**	HI subset	40	35	12	–	16
	All	40	63	13	–	26
**Re-enrolled**	HI subset	–	–	–	16	9
	All	–	–	–	16	41
**Median age (IQR)**	HI subset	44 (31, 53)	38 (31, 55)	27 (21, 39)	50 (31, 58)	45 (34, 56)
	All	”	44 (36, 52)	28 (21, 36)	”	46 (39, 55)
**Female (n, %)**	HI subset	32 (80%)	28 (80%)	6 (50%)	13 (81%)	21 (84%)
	All	”	47 (75%)	7 (54%)	”	52 (78%)
**Median BMI (IQR)**	HI subset					
	All	23 (21, 25)	25 (23, 29)	25 (23, 28)	22 (20, 24)	25 (23, 28)
**Had at least one health condition**	All	6 (15%)	12 (19%)	3 (23%)	3 (19%)	13 (32%)
**Occupation group**						
Critical and emergency	All	5 (13%)	3 (5%)	2 (15%)	2 (13%)	7 (10%)
Paediatric	All	0 (0%)	16 (25%)	1 (8%)	0 (0%)	18 (27%)
Adult medicine	All	6 (15%)	11 (17%)	0 (0%)	4 (25%)	12 (18%)
Surgery	All	2 (5%)	2 (3%)	0 (0%)	1 (6%)	0 (0%)
Allied health	All	0 (0%)	1 (2%)	0 (0%)	1 (6%)	0 (0%)
Support service	All	27 (68%)	30 (48%)	10 (77%)	8 (50%)	30 (45%)
**Vaccination brand**						
GSK	All	10 (25%)	16 (25%)	1 (8%)	1 (6%)	11 (16%)
Sanofi	All	13 (33%)	15 (24%)	11 (85%)	15 (94%)	56 (84%)
Seqirus	All	17 (42%)	32 (51%)	0 (0%)	0 (0%)	0 (0%)
Unknown	All	0 (0%)	0 (0%)	1 (8%)	0 (0%)	0 (0%)

IQR: interquartile range (25th to 75th percentile)

**Table 2:** Linear mixed-effects model estimates for predictors of HI antibody fold-rise by day 14 after influenza vaccination. Fold-rises were modelled on the log scale and back-transformed for interpretation.

Predictor	Estimate (β)	95% CI	P-value
**Intercept**	3.1	2.5–4	
**Prior vaccination**			
5-Prior	reference	—	
1-Prior	1.5	1.1–2.1	0.008
0-Prior	2.6	1.9–3.4	< 0.001
**Year**			
2020	ref	—	
2021	1.3	1.2–1.5	< 0.001
**Log (pre-vaccination titre)**	0.68	0.66–0.71	< 0.001
**A(H3N2) Virus**			
SA19e	reference	—	
Br07	0.84	0.71–0.98	0.032
Pe09	0.76	0.65–0.89	< 0.001
Pe09e	0.55	0.46–0.64	< 0.001
Vi11	0.87	0.74–1	0.083
Vi11e	0.88	0.75–1	0.110
Tx12	1.1	0.9–1.2	0.475
Tx12e	0.81	0.69–0.95	0.011
Sw13	0.5	0.43–0.59	< 0.001
Sw13e	0.58	0.49–0.68	< 0.001
Ka17e	0.4	0.34–0.47	< 0.001
HK14	0.24	0.21–0.29	< 0.001
HK14e	0.87	0.74–1	0.095
Sw17e	0.69	0.58–0.81	< 0.001
Br18	0.32	0.27–0.38	< 0.001
Nc16	0.42	0.36–0.5	< 0.001
Si16e	0.72	0.62–0.85	< 0.001
Brs18	0.48	0.41–0.56	< 0.001
SA19	0.29	0.25–0.35	< 0.001
Da19	0.35	0.29–0.41	< 0.001
HK19e	0.71	0.6–0.83	< 0.001
Ca20	0.23	0.2–0.27	< 0.001
Da21	0.28	0.24–0.33	< 0.001
Da21e	0.37	0.31–0.43	< 0.001
Ma22	0.24	0.2–0.29	< 0.001
**Age**	0.99	0.98–1	0.222
**Sex**			
Female	reference	—	
Male	0.88	0.63–1.2	0.478
**Interaction: prior vaccination x pre-vaccination titre**			
0-Prior x log (pre-vaccination titre)	1	0.97–1.1	0.387
1-Prior x log (pre-vaccination titre)	0.69	0.65–0.73	< 0.001

## Data Availability

De-identified data will be made available under a data-sharing agreement that provides for: (1) a commitment to use the data only for research purposes and not to identify any individual participant; (2) a commitment to secure the data using appropriate computer technology; and (3) a commitment to destroy the data after analyses are completed.
